# The Anterolateral Thigh Perforator Flap in an Innovative Microsurgery Training Method

**DOI:** 10.1097/GOX.0000000000001974

**Published:** 2018-11-13

**Authors:** Parthena I. Deskoulidi, Konstantinos M. Benetatos, Nikolaos A. Maltzaris, Pantelis K. Diamantopoulos, Efthymios D. Basagiannis, Maria D. Kotrotsiou, Spiros D. Stavrianos

**Affiliations:** From the * Department of Plastic Surgery, St. Savvas Cancer Hospital of Athens, Greece; † Department of Plastic Surgery, General Military Hospital of Athens, Greece; ‡ Department of Plastic Surgery, Örebro University Hospital, Sweden; § Department of Plastic Surgery, Evangelismos General Hospital of Athens, Greece.

## Abstract

The road to becoming a good and confident microsurgeon requires love for your work, patience, and good training facilities. Safe and effective training procedures for young microsurgeons during their plastic surgery residency are necessary and should be applied under standardized conditions. We present an innovative microsurgical training method for plastic surgery residents in the operation theater concerning the anterolateral thigh perforator flap (ALT). In a 2-team approach, the ALT flap harvesting begins parallel to tumor resection. Although the tumor excision team still works in the tumor region, and after the reconstructive team has successfully completed the ALT dissection, residents can work distally to the origin of the perforator vessel (which supplies the flap). Their training involves dissection and anastomosis of the continuation of the descending branch, distally to the perforator supplying the flap. Since 2015, eight operations have been performed with this innovative method with the participation of upcoming microsurgeons. A written informed consent is given to all patients. Our study resulted in the improvement of microsurgical skills of the young microsurgeons. There is no impact to the ALT perforator flap or to the operative time. This training procedure can be safely applied as a training method during plastic surgery residency under standardized conditions. We have the joy of seeing our resident’s progress through their high success rates in microsurgery. We recommend this innovative procedure as an adequate teaching method during residency to promote the future of our specialty, and we hope that our students will become even better than their teachers.

## INTRODUCTION

Microsurgical teaching during plastic surgery residency programs may vary in the educational opportunities given to the residents, with regard to microsurgical experience (microsurgery courses). An appropriate training environment requires a high case load, standardized techniques, risk stratification, preselection of patients, and a high level of expertise of the teaching microsurgeon. Economic considerations, safety regulations, a negative perception with an hypothesized prolonged operative time with the resident’s involvement contradict adequate teaching and create obstacles against microsurgery training. Young microsurgeons who have successfully completed the basic and advanced courses in microsurgery (eg, flap dissection courses and video-assisted microsurgery, which are essential for acquiring the high level of technical skill required), are able to perform free flaps in the operation room, as a teaching procedure under standardized conditions.^1–4^ Hirche et al.^1^ showed in their comparative cohort study that microvascular free flaps are a safe and suitable training procedure during structured plastic surgery residency. Their analysis revealed that the anterolateral thigh flap (ALT) was significantly more often performed by residents.^5^

## A NEW TRAINING MODEL TO MAKE YOUNG MICROSURGEONS BETTER

**Fig. 1.**
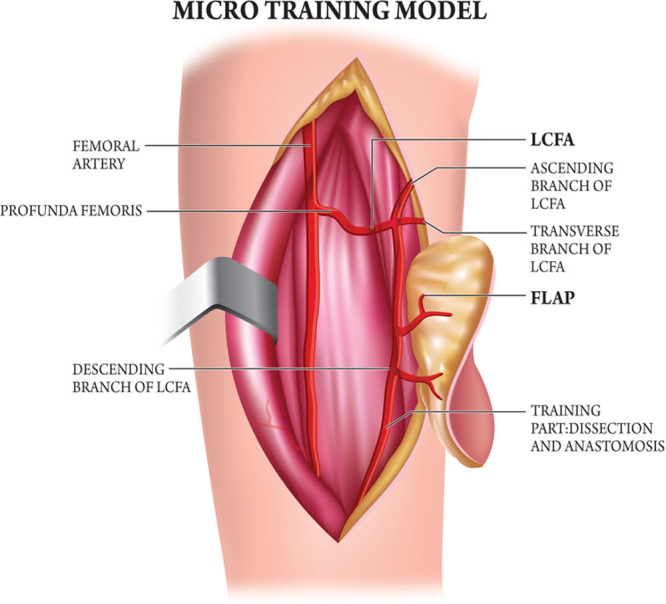
The microsurgical training method.

**Fig. 2.**
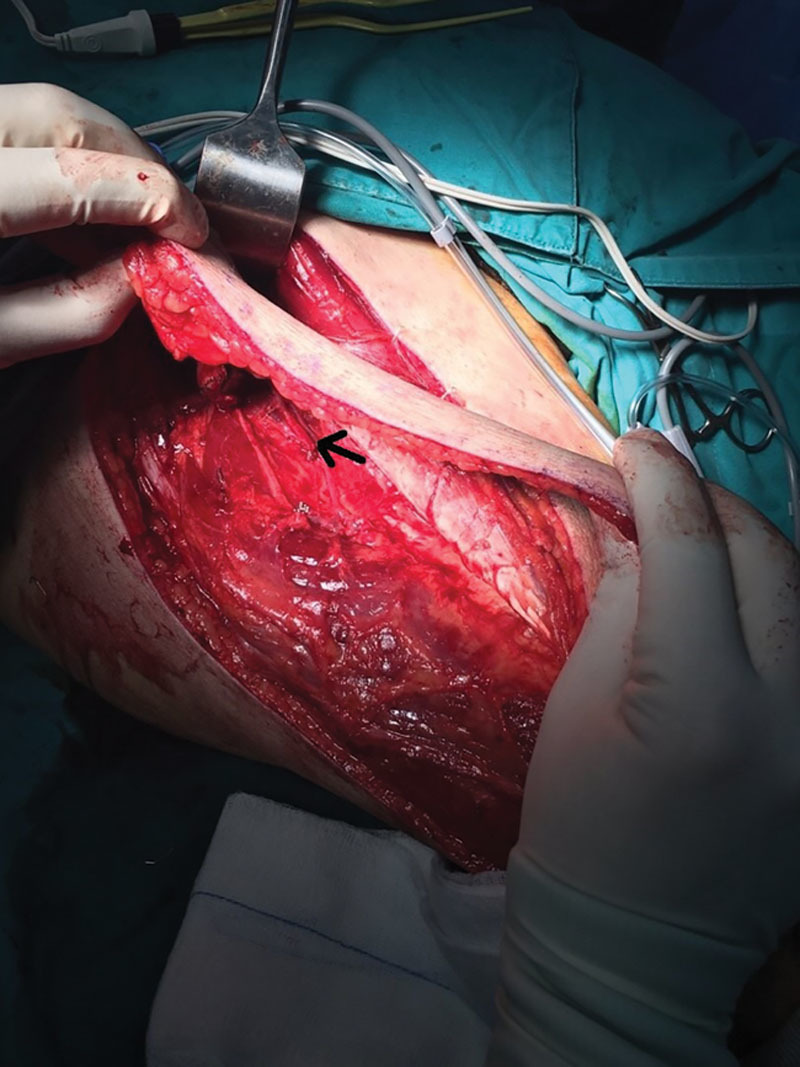
The descending branch which is ligated (educative dissection was performed distally).

**Fig. 3.**
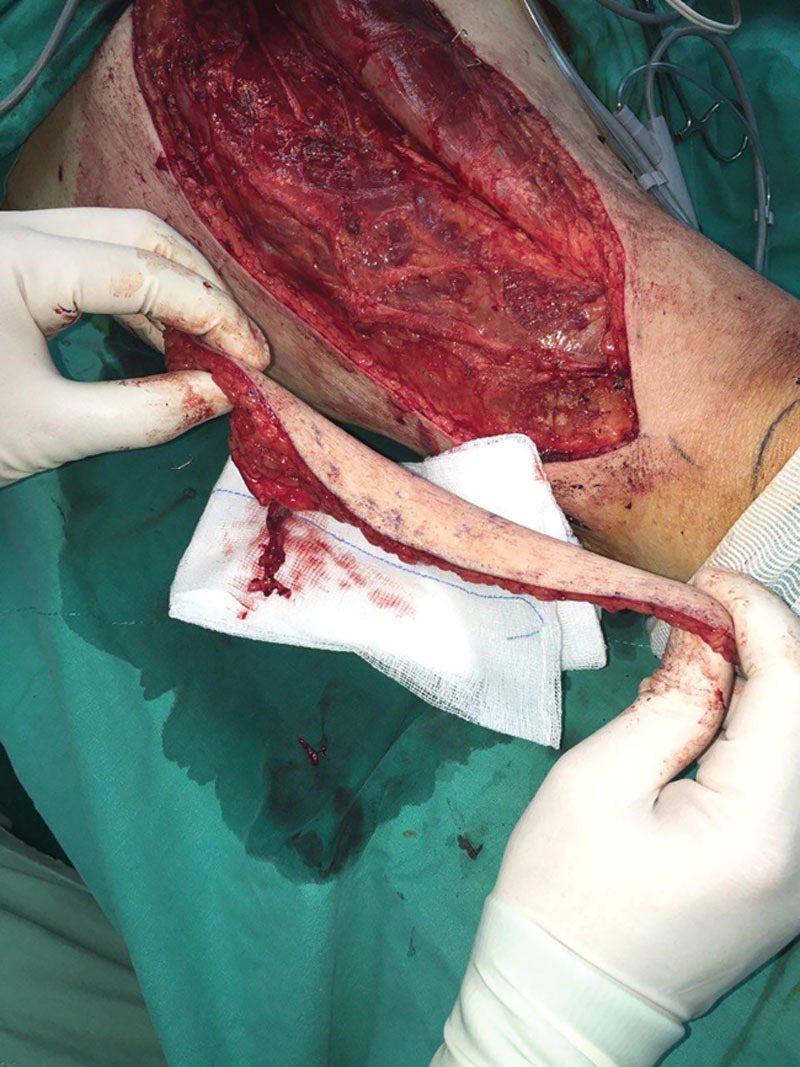
The ALT flap after the educative dissection and ligation of the descending branch (no impact to the flap).

Based on our experience and the cohort studies worldwide, we propose an innovative method in microsurgical training during plastic surgery residency, concerning the ALT. Between 2015 and 2017, 8 procedures have been performed by residents (especially chief residents) with this innovative training model. A written informed consent is given to all patients. The process provide sufficient opportunity to the patient whether to give his consensus or not. Information about the microsurgery method and an explanation about the benefits from this innovative method for young microsurgeons are provided in the informed consent document. The desire of helping the new generation of microsurgeons lead to 8 volunteer patients. In a 2-team approach,^6^ in which the ALT flap harvesting begins parallel to tumor resection, while the tumor excision team still works to the tumor region, and after the reconstructive team has successfully completed the ALT dissection, residents can work distally to the origin of the perforator vessel (which supplies the flap). Hence, they can train in the dissection of the continuation of the descending branch of the lateral circumflex femoral artery, distally to the perforator supplying the flap. While the flap is still in situ and the flow to the continuation of the descending branch is still clamped, just distally to the origin of the perforator, the vessels can be divided and a training anastomosis can be achieved (Figs. 1–3). No nerves implicated and there is no impact to the ALT perforator flap. The time of training is up to 1 hour for 2–3 residents until the tumor resection team completes the resection. If the tumor resection were completed before completion of the training anastomosis, then the descending branch would be ligated, similarly to the standard procedure. Table 1 shows the residents’ involvement in this innovative training model and describes the details of the patient who underwent reconstruction with a free ALT flap. Competence and confidence increased in contrast to new residents who had completed microsurgical courses but did not participate in our innovative procedure.

**Table 1.**
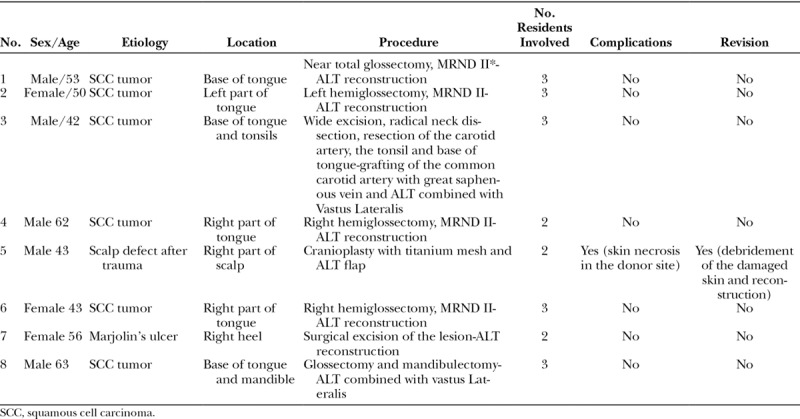
Details of the Patients Who Underwent Reconstruction With a Free ALT With the Innovative Training Model

## CONCLUSIONS

A residency program should offer work-based training opportunities with annual progression toward achieving microsurgical competency. With respect to standardized environmental conditions and risk stratification, we hope that our innovative model of microsurgical training in the operation theater will be helpful for young microsurgeons worldwide and will strengthen their microsurgical skills. The high level of expertise of the teacher, the resident’s experience and the proper institutional infrastructure is mandatory. We recommend this innovative procedure as an adequate teaching method during residency to promote the future of our specialty, and we hope that our students will become even better than their teachers. Giving motivations to young microsurgeons to operate during this safe training method, will reboost the microsurgery specialty and keep it in top and alive.
